# Supplementary material for: Substance and Behavioral Addictions among American Indian and Alaska Native Populations
Source: Int J Environ Res Public Health. 2022 Mar 3;19(5):2974. doi: 10.3390/ijerph19052974 (PMC8910676; doi:10.3390/ijerph19052974)
Supplement: Supplementary file 1 [file ijerph-19-02974-s001.zip › ijerph-1609075-supplementary.pdf]

Supplementary Table S1. Summary Information for Risk Factors Associated with Substance Use Studies.

Risk Factors	Author(s)	Study Aim	Study Design	Sample Size	Key Findings	Measures Used
Historical Trauma	Brave Heart, 2003	Review conceptual framework of historical trauma and historical trauma response, discuss measurement of trauma, and innovations to address historical trauma	Review		Deeper study into historical trauma (e.g., prevalence, characterstics is needed). Greater use of historical trauma scales is warranted to increase knowledge. Indigenous practices can inform evidence-based treatments There is also a need to diversity interventions in terms of implementation levels	
	Kirmayer et al., 2014	Compare historical events of massive, organized violence to identify common mechanisms and distinctive features	Review		Historical trama is complex and experienced differently by diverse populations. Structural problems compound trauma and differ generationally. Understanding historical trauma requires a broad understanding of the multiple factors that contribute to it.	
	Gone et al., 2019	Examine the theoretical foundations of historical trauma among Native Americans	Systematic review		Several studies reported statistically significantly relationships between indicators of historical trauma and adverse health outcomes. Historical trauma can potentially help explain modern day Indigenous health problems and needs further study	

	Brave Heart et al., 2011	Review the historical trauma framework, research, and current innovations aimed at addressing historical trauma among AIANs	Review	Culturally responsive interventions for grief resolution and trauma are necessary to reduce emotional suffering. Research on the best strategies for this work should be guided by Indigenous people. .
	Brown-Rice et al., 2013	Examine the theoretical foundations of historical trauma Among Native Americans	Review	Native Americans experience historical loss symptoms like depression, substance dependence, diabetes, unemployment. Trauma has an intergeneration impact on physiological functioning
	Evans-Campbell, 20008	Presents a multilevel framework for exploring the impact of historically traumatic events	Review	Key characteristics of historical trauma include widespread collective distress and mourning.. Current life stressors align with the context of historical trauma.
	Mohatt et al., 2014	Discuss the connection between historical trauma and present-day experiences	Review	Framing historical trauma as a public narrative puts more emphasis on the development and impact of present-day events and their connection to the past
	Morgan & Freeman, 2009	Examine treatments focused on cultural strengths for Alaska Natives	Review	Historical and intergenerational trauma are strong contributors to substance use.

	Nutton & Fast, 2015	Discusses the relationship between historical trauma attributed to colonial policies and rates of substance use among AIAN populations	Review		Historical trauma has been associated with higher rates of substance use among AIANs compared to other racial groups. Decolonizing strategies, identity development, and culturally relevant interventions are needed to mitigate the negative effects of historical trauma	
	Ehlers et al., 2013	Evaluate the relationship between thoughts of historical loss and its symptoms to current traumatic events, cultural identifications, and mental disorders	Cross-sectional	N=306 AI Adults	Anxiety/affective disorders and substance dependence were correlated with historical loss Participants reported thinking about historical losses and symptoms of distress	Alcoholism: Semi-Structured Assessment for the Genetics of Alcoholism (SSAGA) Historical loss: Historical Loss Scale, Historical Loss Associated Symptoms Scale Trauma: Stressful-Life-Events Scale Cultural Indentification: Orthogonal Cultural Identification Scale (OCIS)

Risk Factors	Author(s)	Study Aim	Study Design	Sample	Key Findings	Measures Used
	Soto et al., 2015	Examine the relationship between ethnic identity, participation in cultural activities, and stressful life events to smoking	Cross-sectional	N=969 AI Youth	Historical trauma mediated the associations of ethnic identity, cultural activities, and stressful life events with smoking. Stronger ethnic identity predicted greater past-month smoking.	Ethnic Identity: Multi-group Ethnic Identity Measure (MEIM) Stressful life events: Multicultural Events Schedule for Adolescents (MESA) scale Cultural activities: 2 items, "Have you ever participated in a drum circle/ sweat lodge" Historical trauma: Historical Loss Scale
	Whitbeck et al., 2004	Discuss the development of two measures relating to historical trauma among AI individuals	Qualitative (focus groups)	N=24 AI Elders	The Historical Loss Scale and Historical Loss Associated Symptoms Scale show high internal reliability. Current generations of AI adults have frequent thoughts relating to historical trauma and experience.	Historical Trauma: Historical Loss Scale, Historical Loss Associated Symptoms Scale
	Wiechelt et al., 2012	Examine the impact of historical trauma on substance misuse and family cohesion	Cross-sectional	N=120 AI Adults	Urban AIs reported experiencing more historical trauma compared to reservation-based AIs. Historical trauma symptoms are associated with past month alcohol use, lifetime use of illicit drugs, and lower family cohesion.	Historical Trauma: Historical Loss Scale, Historical Loss Associated Symptoms Scale

Sociocultural Risk Factors	McKinley et al., 2021	Assess alcohol use from a sex-specific wellness approach	Cross-sectional	N = 489 AIAN	Males have 3 times higher drinking rates compared to females. Positive relationship between alcohol and depressive symptoms.	Alcohol use (created for the study), Body Mass Index (BMI), Self-rated Abilities for Health Practices Scale, Patient Health Questionnaire- 9
	Kulis et al., 2006	Examine the social contexts of AI youth encounters with drug offers and their relationship to substance use	Cross sectional	**N=71 AI Youth**	**Exposure to drug offers through relatives or peers was associated with substance use. For females, exposure through parents was a significant predictor of future substance use**	Ecosystemic context of drug use: The Problem Situations Inventory for American Indian Youth
	Kulis et al., 2002	Examine how strength of ethnic identity, multiethnic identity, and other indicators of biculturalism relate to drug use norms of urban AIAN middle schoolers	Cross sectional	N=434 AIAN	Higher levels of ethnic pride and greater academic achievement were positively related to antidrug norm adherence. .	Drug use norms: 26 item questionnaire developed for the study using factor analysis
	Whitesell et al., 2012	Study the epidemiology and etiology of substance use among AIANs	Review		Patterns of substance use are diverse. Alcohol directly impacts physical and mental health	
	Campbell et al., 2015	To assess the acceptability of Therapeutic Education System to address substance use.	Cross-sectional (Mixed Methods)	N= 40 AIAN	Therapeutic Education System was acceptable among this AIAN sample; however, adaptations to AIAN culture could improve adoption.	Self-report substance use: Timeline Follow-Back method; Cultural/ethnici identity: The Scale of Ethnic Experience; Intervention acceptability: Qualitative interviews
**Risk Factors**	**Author(s)**	**Study Aim**	**Study Design**	**Sample**	**Key Findings**	**Measures Used**

	McKinley et at,. 2021	Examine AI alcohol use from a sex-specific wellness aproach and identify risk and protective factors	Cross-sectional (qualitative)	N=489 AI Adults	Nicotine dependence significantly associated with SUD. Association between depression and smoking is bidirectional and positive. Strong association between lifetime nicotine dependence and mood/anxiety disorders among AIANs	Alcohol use disorder: Alcohol Use Disorder and Associated Disabilities Interview Schedule DSM-IV (AUDADIS-IV) Lifetime nicotine dependence: AUDADIS-IV Lifetime and current psychiatric disorders: AUDADIS-IV<br><br>Data taken from the NESARC
Poverty and Environment	Whitesell, at al.,2012	Describe the epidemiology and etiology of substance use within the AIAN community	Review		Alcohol impacts both physical and mental health within all cultural groups in AIAN populations. Risk and protective factors span across individual, social, and cultural categories.	
	Walters et al., 2002	Propose a new stress-coping model for AIANs	Review		"Indigenist" perspective incorporates impact of historical trauma and ongoing oppression, rather than the Eurocentric paradigm which focuses on individual pathology. Many AIAN substance use issues are directly related to colonization status and various sources of discrimination.	

Davis at al., 2016	Analyze contemporary AI poverty and its rooms in geography and labor market opportunities	Labor market analysis	N= 110,644 AIAN(American Community Survey)	Tribal-owned casinos reduce poverty, but not significantly enough to compensate for the poverty disparity between AIAN and whites. AIAN poverty persists regardless of labor market opportunities due to history of government-sponsored control and discrimination.	American Indian Poverty: 2011 American Community Survey
Jones 2008	Identify features in domestic violence in an AIAN sample and identify needs and barriers to service seeking and delivery.	Observational	N= 41	Domestic violence may be associated with historical trauma, poverty, drugs, alcohol, and rural isolation.	Interview guide developed for the study
Lafromboise et al., 2006	Identity the factors that influence resilience for AIAN adolescents	Cross-sectional	N=212 Youth	Primary risk factor for future problem behaviors was perceived discrimination. Protective factors include having a supportive mother, perceived community support, and higher levels of enculturation.	Self-esteem: Tri-Ethnic Center's self-esteem scale Enculturation: 20 item scale measuring involvement in traditional activities, identification with AIAN culture, and traditional spiritual involvement Identification with AI Culture: American Indian Cultural Identification Scale Maternal warmth: 6 item scale Community support: 3 item index Perceived discrimination: 10 item scale Resilience: multidimensional construct involving school attitude, academic plans, use of drugs, etc.

	Castor et al., 2006	Examine the health status of AIAN populations served by federally-funded Indian Health organizations	Cross-sectional	N=51 AIAN Health Organizations	AIAN populations aremore likely have a lower socioeconomic status compared to the general population. Large health disparity observed in maternal/child health with AIAN mothers receiving less prenatal care and higher rates of alcohol use.	National data: 2000 US Census Death certificate: 1990-1999 (National Center for Health Statistics) Birth certificate: 1991-2000 (National Center for Health Statistics) Infant death/natality: 1995-2000 (National Center for Health Statistics)
	Szlemko et al., 2006	Outline the prevalence of alcohol misuse among AIANs, discuss interventions, treatment, and risk factors	Review		AIAN adults and adolescents have higher rates of alcohol useand fetal alcohol syndrome compared to other U.S. ethnic groups. Risk factors include poverty, gender, family history, peer attitudes, parent monitoring, and acculturation. Culturally specific interventions are most effective.	
	Peterson et al., 2002	Understand the life experiences and perceived recovery needs of AIAN women in substance abuse treatment programs	Cross-sectional (qualitative and quantitative)	N=9 IHS-Funded Treatment Centers N=52 Staff Members N=60 AIAN	Strongest treatment motivation was to maintain or regain custody of their children. Nearly all women used drugs or alcohol to suppress emotions (e.g., grief, pity, and loneliness). Women reported disliking the strict regimens and rules of treatment programs and cited them as reasons for leaving/avoiding treatment.	History of AOD use, social and behavioral risk factors, and duration of treatment information was obtained from participants' medical records

Risk Factors	Author(s)	Study Aim	Study Design	Sample	Key Findings	Measures Used
	LaFromboise et al., 2003	Review the assessment and treatment of AIAN children and adolescents	Review		Increase in suicide and suicide-related behaviors within AIAN communities after colonization. Ecological risk factors include historical trauma, acculturation stress, pervasive poverty, community violence, etc. Individual risk factors include depression, substance abuse, anxiety, stress, psychiatric disorders, etc.	
	Espel at al., 2015	Provide background information from studies that examined risk and protective factors or suicide prevention programs for AIAN youth	Annotated bibliography	N=31	Risk factors for AIAN adolescents include alcohol abuse, suicide clustering, intergenerational trauma, and enculturation. Protective factors for AIAN youth include school experience, caring families, and supportive tribal leaders. Importance of cultural relevance and community involvement in developing prevention/treatment programs.	

Risk Factors	Author(s)	Study Aim	Study Design	Sample	Key Findings	Measures Used
Biological	Beauvais, 1998	Outline the historical and modern factors that contribute to the high prevalence of alcohol use among AI	Review		Early demand, lack of regulation, and strong encouragement of alcohol use during colonialism may have contributed to a "tradition" of heavy use. AI youth drink more heavily and experience more negative consequences compared to non-AI peers. Alcohol abuse has greater health consequences for the AI population due to heavier levels of drinking, despite potentially higher rates of abstainers compared to non-AI groups.	
	Mulligan at al., 2003	Determine the prevalence of alcohol metabolism enzymes in a sample of AI	Cross-sectional	N=90 AI (clinical interviews)<br><br>N=499 AI (provided blood samples)	Allelic variants (ADH1B, ALDH2) have been identified as altering alcohol metabolism in humans and providing protection against alcoholism. Neither of the alleles were detected in the study population suggesting an additional genetic factor that increases AI vulnerability to alcohol dependence.	Alcohol dependence: Schedule for Affective Disorders and Schizophrenia-Lifetime Version (SADS-L) with probes added using the Research Diagnosis Criteria and DSM-III-R

	Spillane et al., 2020	Examine trends in the rates of alcohol-induced deaths among U.S. population subgroups	Observational	N=425,045	Large increases in alcohol-induced mortality were observed among both men and women. Significant increases in mortality were observed among all racial/ethnic groups from 2013 to 2016.	Death Certificates
Mental Health	Beckstead et al., 2015	Examine pre to post-change of patients in a substance use residential treatment that incorporated Dialectic Behavior Therapy with specific cultural/spiritual practices	Cross-sectional	N=229 AIAN Youth	96% of the adolescents either "recovered" or "improved" using clinical significance change criteria. Support for integrating Western and traditional methods of care when addressing SUD and other mental disorders in AIAN adolescents.	Youth Outcome Questionnaire-Self Report (YOQ-SR)
	Moghaddam et al., 2014	Examine the comorbidity between nicotine dependence and psychiatric and SUD among AIANs	Secondary data analysis	N=701 AIAN	Nicotine dependence significantly associated with SUD. Association between depression and smoking is bidirectional and positive. Strong association between lifetime nicotine dependence and mood/anxiety disorders among AIANs.	Alcohol use disorder: Alcohol Use Disorder and Associated Disabilities Interview Schedule DSM-IV (AUDADIS-IV) Lifetime nicotine dependence: AUDADIS-IV Lifetime and current psychiatric disorders: AUDADIS-IV

	Warne et al., 2017	Assess the prevalence of ACEs and their association with behavioral health in AIAN and non-AIAN populations	Cross-sectional	N=7,675 survey respondents; 516 AIANs	AIANs had a higher prevalence of abuse, neglect, and household dysfunction compared to non-AIANs For all groups, having six or more ACEs increased the risks of depression, PTSD, anxiety, severe alcohol misuse, and smoking	Adverse childhood experiences: Ten categories of ACEs Mental health conditions: Patient Health Questionnaire (PHQ-2), General Anxiety Disorder scale (GAD-2), Primary Care PTSD Screen (PC-PTSD) Alcohol and tobacco use: Alcohol Use Disorders Identification Test (AUDIT-C), Behavioral risk Factor Surveillance System questionnaire (BRFSS)
	Brave Heart et al., 2016	Explain historical trauma theory and offer specific Native examples of the historical trauma response	Review		Modern psychosocial problems of Native people should be viewed within the context of historically traumatic losses •Quality of parenting is a large risk/protective factor for substance use	
	Manson et al., 2000	Summarize data on mental health needs and mental health care of AIANs	Review		Limitedservice utilization observed for AIANs and other social minorities experiencing mental health disorders Suicide is more common among male AIAN adolescents,	

	Baldwin et al., 2011	Investigate risk and protective factors related to alcohol and drug use among American Indian youth	Cross-sectional	N total=221 AI Youth	Relationship between stressful life events and depressed mood, substance use, and other risky behaviors<br>No direct relationship between cultural identity and substance abuse/cultural identity as protective<br>Strong relationship between AI identification and protective family/peer influences	Age<br>Stressful life events: 12 item scale ("did these events happen to you")<br>Social support: Multidimensional Scale of Perceived Social Support<br>Protective family and peer influence<br>Native American identity: Orthogonal cultural identification scale<br>White/Anglo identity: Orthogonal cultural identification scale<br>Depressed mood: CES-D<br>Substance use: Tri-Ethnic Center for Prevention<br>Risky behavior: questions adapted from Alexander et al. 1995
	Eitle et al., 2014	Examine the role of coping strategies in moderating the stress-substance use association for AI adolescents	Cross-sectional (qualitative survey)	N=568 Youth (White and AI)	Problem-focused coping strategies were protective for substance use among both white and AI respondents<br>Self distraction, an avoidant coping strategy, was associated with increased risk of marijuana use for AI adolescents but not other racial minority groups	Coping measures: Brief COPE<br>Recent life events: 26 items, "has this happened to you in the past year"<br>Chronic strains: 10 items capturing stressful conditions related to parents/school/relationships/general

	Rieckmann et al., 2012	Examine substance use, mental health disorders, and treatment needs for AIANs with SUD	Cross-sectional (qualitative and quantitative)	N=74 urban AIAN N=121 reservation-based AIAN	Urban AIs more likely to report employment problems, polysubstance use, and history of abuse Reservation-based AIs reported having more severe medical problems and prevalence of psychiatric problems	Addiction: Addiction Severity Index (ASI)
	O'Connell et al., 2006	Define patterns of alcohol use among two AIAN communities and examine associations between alcohol use and other indicators of health status	Secondary data analysis (quantitative)	N=1287 AI Adults	Drinkers in the highest risk category for alcohol dependence were more likely to report drug use disorders, mood/anxiety disorders, alcohol-related physical disorders, and lower quality of life High-quantity, yet low frequency pattern of drinking identified for AIANs For females, high-quantity, frequent drinking was associated with drug use disorders For males, high-quantity, frequent drinking was associated with mood/anxiety disorders	Alcohol use: quantity and frequency of alcohol drank Alcohol, drug, and other psychiatric disorders: University of Michigan version of the Composite International Diagnostic Interview (UM-CIDI) Physical health problems: self-reported diagnosis of medical conditions Health-related quality of life: Medical Outcomes Study Short Form-36 (SF-36)
**Risk Factors**	**Author(s)**	**Study Aim**	**Study Design**	**Sample**	**Key Findings**	**Measures Used**
Polysubstance Use						

	O'Conell et al., 2007	Examine childhood characteristics associated with stage of substance use in adulthood in two AI populations	Cross-sectional	N=2070 AI Adults	Strong association between experiencing a traumatic event and each stage of substance use<br>Higher level substance use associated with lifetime substance use dependence, other adult psychiatric disorders, poorer physical health, and legal problems	Stage of substance use: 4 hierarchical stages<br>Traumatic experiences: witnessing trauma, family violence, etc.<br>Conduct problems: list of 12 negative and violent behaviors<br>Substance use: age of initiation
	Whitbeck et al., 2008	Investigate changes in prevalence rates for mental and substance use disorders for indigenous adolescents	Longitudinal	N=651 Indigenous Youth	Increase in prevalence rates for SUD and conduct disorders from 10-12 years to 13-15 of age<br>AI adolescents had higher overall prevalence rates of lifetime conduct disorders and lifetime substance abuse disorders compared to the general population	Substance abuse disorders: Diagnostic Interview Schedule for Children-Revised (DISC-R)<br>Adult caretaker diagnosis: University of Michigan Composite International Diagnostic Interview (UM-CIDI)
	Dickerson et al., 2009	Examine comorbidity of nicotine dependence and substance use disorders among AIANs	Cross-sectional	N=480 AI Adults	Lifetime nicotine dependence was associated with alcohol and drug use disorders, anxiety/affective disorders, PTSD, pathological gambling, and antisocial personality disorder	Psychiatric disorders: Quick-Diagnostic Interview Schedule (Q-DIS)<br>Nicotine dependence: Diagnostic and Statistical Manual of Mental Disorders (DSM-IV)
**Risk Factors**	**Author(s)**	**Study Aim**	**Study Design**	**Sample**	**Key Findings**	**Measures Used**
Family Risk Factors	Burnette et al., 2016	Review available research on culturally specific risk and protective factors	Systematic review	N= 51 studies with AIAN Youth	There is an overlap of risk factors on mental health and substance use which contribute to disparities	

	Burnette et al., 2016	Provide a culturally relevant framework to explain, predict, and prevent violence experienced by AIAN individuals	Cross-sectional	N=49 Indigenous Women	Individuals who have been affected by violence have experienced familial divsions, absent parents, family substance use, and limited bonding	
	Unger at el., 2020	Determine if cultural factors such as spirituality and ethnic identity are associated with substance use in AIAN adolescents	Cross-sectional	N=156 AIAN Youth	Strong ethnic identity was protective against marijuana, alcohol, and cigarette use, Spirituality was associated with an increased risk of marijuana and cigarette use Previous use of traditional tobacco for ceremonial purposes was not associated with past-month substance use	Past-month use of substances: Youth Risk Behavior Surveillance Survey Spirituality: Spirituality Scale Ethnic identity: Multigroup Ethnic Identity Measure
	Hurdle et al., 2003	Understand familial influences on substance use by AIAN kinship.	Cross-sectional	N=19	Kinship members exert influence on other family members with regard to high-risk behaviors.	Semi-structured interview guide
	Warne et al., 2015	Discuss health disparities found in AIAN populations and how they relate to various psychosocial influences	Review		Historical trauma, adverse childhood and adult experiences, contribute to chronic disease disparities and poor health outcomes and impact parenting abilities for future generations	

	Olson et al., 2020	Review literature on substance abuse studies focused on AIAN populations in the context of historical trauma from boarding school experience and loss of family relationships	Systematic review	N=27 studies	Boarding school residents reported a profound loss of cultural identity PTSD, substance use, and grief associated with boarding school attendance Children who attended boarding schools reported few positive parenting experiences and difficulty with identifying with cultural, collectivist values	
	Felitti et al., 1998	To examine the relationship betweeen adverse childhood experiences and leading causes of death in adult life	Cross-sectional survey	N= 13,494 representative sample	More than 50% of the sample had at least one adverse experience, and 25% reported having more than two of these experiences. A positive relationship between the number of experiences and adult illnesses was observed.	Health appraisals including, health history and physical examinations by medical providers.

Supplementary Table S2. Summary of Information for Protective Factors associated with Substance Use Studies.

Protective Factors	Author(s)	Study Aim	Study Design	Sample	Key Findings	Measures Used
	Cwik at el., 2019	Discuss the process of developing and implementing an intervention	Intervention pilot	N= 1,400 Youth	Community-based interventions may help teach cultural traditions, belongingness, and cultural identity to AI youth<br>Expertise from Apache Elders can help build interventions for youth	
**Community Factors**	Lewton et al., 2000	Explain how Navajo principles are understood, demonstrated, and elaborated in three different healing traditions	Qualitative (narrative-based interviews)		Sa'ah naagháí bik'eh hózh specifies that conditions for health are harmonious with the physical/spiritual world<br>Traditional healing encourages effective engagement, proper family relations, understanding of one's cultural and spiritual histories, and use of kinship to establish bonds	
	Moore et al., 2010	Explore the effects of Native-focused, peer recovery support programs			Firestarter program incorporates indigenous helpers in encouraging and supporting sobriety and wellness for AIAN members<br>Training of local peer leaders helps develop community capacity	
	Whitbeck at al., 2012	Review literature on current AIAN substance abuse prevention programs	Review		There are numerous prevention programs originating from AIAN communities themselves. There might need to be a	

					paradigm shift from adapting European American prevention science programs to creating cultural best practices from AIAN communities	
	Park et al., 2012	Estimate risk differences of overdoses among AIANs	Cross-sectional	N= 2,031	U.S. born AIANs have the highest incidence of overdoses	
	Kirmayer et al., 2011	Redefine resilience research by incorporating indigenous constructs			Frame Indigenous constructs in terms of processes that foster resilience and involve indivduals, communities, and larger systems	
**Protective Factors**	**Author(s)**	**Study Aim**	**Study Design**	**Sample**	**Key Findings**	**Measures Used**
Family Factors	Wang et al., 2011	Examine the relationship between family management practices and early adolescent problem behavior	Cross-sectional (qualitative)	N=1,030 Representative sample including AIsYouth	Adolescent antisocial behaviors and substance use increased over time. Positive behavioral engagement decreased. Increases in adolescents' age was associated with diminished parental rule making, knowledge, and support. Parental warmth determined the level of association between parental knowledge and adolescent problem behaviors	Adolescent antisocial behavior: Teacher Perception of Risk (TRISK; Soberman, 1994) Substance use: Student Self-Report Survey (SSRS; Dishion & Stormshak, 2001) Positive behavioral engagement: Social Skills Rating System (Gresham & Elliott, 1990) Parental knowledge: 4 item scale Parental rule making: 4 item scale Parental use of positive reinforcement: 6 item assessment

Supplementary Table S3. Summary of Information for Behavioral Addictions.

Protective Factors	Author(s)	Study Aim	Study Design	Sample	Key Findings	Measures Used
	Cozzetto et al., 1996	Examine relationship between the increase in tribal-owned casinos and pathological gambling activities	Region Case Study	N = 1,204	Tribal sovereignty led to an increase in tribal-owned casinos and high stakes gambling	
	Volberg et al., 2006	Assess the scope of probematic gambling in New Mexico	Technical report<br><br>Cross-sectional (qualitative)	N=2,850 Total, N=589 AIAN Specific	Larger portion of AIANs have gambled in the past year compared with non-AIANs (73% vs. 67%)<br>Prevalence rate of at-risk, problem and pathological gambling among AIANs in New Mexico is higher than among non-AIANs	Problem gambling: NORC DSM-IV Screen for Gambling Problems (NODS), Canadian Problem Gambling Index (CPGI)
Gambling	Westermeyer et al., 2005	Examine the prevalence of pathological gambling and it's relationship to physical/mental disorders	Cross-sectional (qualitative)	N=1,624 AI and Hispanic Adults.	Pathological gambling was associated with substance, mood, and antisocial personality disorder<br>AI veterans had a 10% lifetime prevalence of pathological gambling	Clinical conditions: Quick-Diagnostic Interview Schedule (Q-DIS), Brief Symptom Index (BSI-57), Michigan Assessment-Screening Tool/Alcohol-Drug (MAST/AD), Posttraumatic Stress Checklist (PCL)<br>Lifetime gambling and pathological gambling: Quick-Diagnostic Interview Schedule (Q-DIS)
	Patterson-Silver Wolf et al., 2014	Examine the patterns of gambling and alcohol abuse among AIANs	Cross-sectional	N=415 AIANs	AIANs have a higher rate of alcohol abuse and the rate of gambling is twice as high as the general population.	Survey created for this study. Diagnostic Interview Schedule, Canadian Problem Gambling Index, South Oaks

					Gambling Screen, Native American Identity Scale
Dowling et al., 2017	Identify early risk and protective factors associated with subsequent development of gambling problems longitudinally	Systematic review	N=23 articles	Individual risk factors for problem gambling include alcohol use frequency, antisocial behavior, depression, male gender, cannabis use, illicit drug use, impulsivity, number of gambling activities, problem gambling severity, sensation seeking, tobacco use, violence, undercontrolled temperament<br>Protective factors include socio-economic status, parent supervision, and social problems	
Potenza et al., 2019	Provide an overview of gambling disorder, it's prevalence within the U.S., neurobiology, and available treatment programs	Review		Gambling revalence across the U.S. ranges from 0.4% to 0.6%<br>Risk factors include having low levels of formal education, history of trauma, psychopathology, and impulsivity<br>Protective factors include parental supervision and religious attendance	
Volberg et al., 1997	Compare the literature on gambling and problem gambling among indigenous groups in New Zealand	Cross-sectional (qualitative)	N=3,933	Gambling-related problems, expenditures, and involvement are higher among indigenous respondents	Gambling:Created for the study-asked "have you tried this type of gambling in the past year/6 months?", South

	and in North Dakota			compared to non-Indigenous respondents	Oaks Gambling Screen
Welte et al., 2017	Examine correlates of problem gambling and potential etiology	Cross-sectional (qualitative)	N=2,963 respondents from a telephone survey that was conducted across the U.S.	Males, people of color with less formal education, and those between 31-40 years old had the highest average problem gambling symptoms<br>Religiosity was associated with less problematic gamblin<br>Impulsivity and depression were associated with problem gambling	Scale developed for the study by combining the Diagnostic Interview Schedule (DIS), the South Oaks Gambling Screen Revised (SOGS-R) and the Canadian Problem Gambling Index (CPGI)
Barnes et al., 2017	Examine gambling risk factors and problem gambling	Self-report survey	N total= 3,474 general U.S. population<br>N=549 Native Americans	Neighborhood disadvantage adverly impacts overall gambling	Frequent gambling: defined by frequency of 15 types of gambling<br>Problem gambling: Diagnostic Interview Schedule, South Oaks Gambling Screen, and Canadian Problem Gambling Index
Dion et al., 2015	Investigate the link between child sexual abuse, residential school attendance,, and pathological gambling	Cross-sectional	N=358 AIAN Adults	Sexual abuse and residential school attendance were associated with pathological gamling	Pathological gambling: South Oaks Gambling Screen (SOGS)<br>Child sexual assault: "have you been sexually assaulted before age 18"<br>Residential schooling: "have you attended a residential school"
Kong et al., 2016	Examine the association between problem-gambling	Secondary data analysis	N=43,093; N= 679 AIANs (National Epidemiologic Survey on	Problem-gambling severity was higher among AIAN adults than	Axis-I/Axis-II diagnostic categories: Alcohol Use Disorder and

	and psychiatric disorders.		Alcohol and Related Conditions)	among Caucasian adults Problem-gambling severity was associated with psychiatric disorders in both AIAN and Caucasian adults	Associated Disability Interview Schedule-DSM-IV (AUDADIS-IV) Problem-gambling: AUDADIS-IV Data taken from the National Epidemiological Survey on Alcohol and Related Conditions (NESARC)
